# Atheroprotection through SYK inhibition fails in established disease when local macrophage proliferation dominates lesion progression

**DOI:** 10.1007/s00395-016-0535-8

**Published:** 2016-02-18

**Authors:** Alexandra Lindau, Carmen Härdtner, Sonja P. Hergeth, Kelly Daryll Blanz, Bianca Dufner, Natalie Hoppe, Nathaly Anto-Michel, Jan Kornemann, Jiadai Zou, Louisa M. S. Gerhardt, Timo Heidt, Florian Willecke, Serjosha Geis, Peter Stachon, Dennis Wolf, Peter Libby, Filip K. Swirski, Clinton S. Robbins, William McPheat, Shaun Hawley, Martin Braddock, Ralf Gilsbach, Lutz Hein, Constantin von zur Mühlen, Christoph Bode, Andreas Zirlik, Ingo Hilgendorf

**Affiliations:** Department of Cardiology and Angiology I, University Heart Center Freiburg, Hugstetter Str. 55, 79106 Freiburg, Germany; Spemann Graduate School of Biology and Medicine, University of Freiburg, Freiburg, Germany; Faculty of Biology, University of Freiburg, Freiburg, Germany; Department of Medicine, Brigham and Women’s Hospital, Harvard Medical School, Boston, MA USA; Center for Systems Biology, Massachusetts General Hospital, Harvard Medical School, Boston, MA USA; Peter Munk Cardiac Centre, Toronto, ON Canada; AstraZeneca R&D, Molndal, Gothenburg, Sweden; AstraZeneca R&D, Alderley Park, Macclesfield, UK; Institute of Experimental and Clinical Pharmacology and Toxicology, University of Freiburg, Freiburg, Germany

**Keywords:** Atherosclerosis, SYK, Monocytes, Macrophages, Progenitors, Proliferation, Egress

## Abstract

Macrophages in the arterial intima sustain chronic inflammation during atherogenesis. Under hypercholesterolemic conditions murine Ly6C^high^ monocytes surge in the blood and spleen, infiltrate nascent atherosclerotic plaques, and differentiate into macrophages that proliferate locally as disease progresses. Spleen tyrosine kinase (SYK) may participate in downstream signaling of various receptors that mediate these processes. We tested the effect of the SYK inhibitor fostamatinib on hypercholesterolemia-associated myelopoiesis and plaque formation in Apoe^−/−^ mice during early and established atherosclerosis. Mice consuming a high cholesterol diet supplemented with fostamatinib for 8 weeks developed less atherosclerosis. Histologic and flow cytometric analysis of aortic tissue showed that fostamatinib reduced the content of Ly6C^high^ monocytes and macrophages. SYK inhibition limited Ly6C^high^ monocytosis through interference with GM-CSF/IL-3 stimulated myelopoiesis, attenuated cell adhesion to the intimal surface, and blocked M-CSF stimulated monocyte to macrophage differentiation. In Apoe^−/−^ mice with established atherosclerosis, however, fostamatinib treatment did not limit macrophage accumulation or lesion progression despite a significant reduction in blood monocyte counts, as lesional macrophages continued to proliferate. Thus, inhibition of hypercholesterolemia-associated monocytosis, monocyte infiltration, and differentiation by SYK antagonism attenuates early atherogenesis but not established disease when local macrophage proliferation dominates lesion progression.

**Electronic supplementary material:**

The online version of this article (doi:10.1007/s00395-016-0535-8) contains supplementary material, which is available to authorized users.

## Introduction

Atherosclerosis involves the accumulation of lipids and inflammatory cells in the arterial intima forming plaques. Macrophages ingest lipids and elaborate inflammatory mediators that promote plaque progression and destabilization [1]. During atheroma initiation, macrophages accumulate primarily through infiltration of Ly6C^high^ monocytes. In established plaques, however, macrophages renew through local proliferation rather than recruitment [13]. It is unknown whether prolonged interference with monocyte recruitment to established plaques can limit macrophage accumulation.

Spleen tyrosine kinase (SYK), a cytosolic signaling protein, binds to various receptors and adaptors containing immunoreceptor tyrosine-based activation motifs (ITAM). Integrin signaling in myeloid cells, for example, requires the ITAM-bearing DAP12 adaptor and subsequent SYK activation to induce cell adhesion [9, 16]. We showed that treatment with the orally available SYK inhibitor fostamatinib reduced inflammatory cell recruitment to the vessel wall and infiltration to sites of inflammation in low density lipoprotein receptor deficient (Ldlr^−/−^) mice. Consequently, these animals developed less atherosclerosis [5]. Of note, fostamatinib demonstrated significant improvement in ACR20 scores over placebo in two recent phase 3 trials with rheumatoid arthritis (RA) patients [5, 17]. The long-term effects of this SYK inhibitor on cardiovascular events in this high risk study population with chronic inflammation and accelerated atherosclerosis [4, 14] remains unknown.

The present study compared the efficacy of SYK inhibition in early and established atherosclerosis. We used Apoe^−/−^ mice, which develop highly inflammatory atherosclerosis characterized by hypercholesterolemia-associated monocytosis [15]. The myelopoietic growth factors GM-CSF and IL-3 stimulate monocyte production in atherosclerosis [12]. Their receptors share a common beta chain (CBS) that associates with SYK [18]. We hypothesized that SYK inhibition by fostamatinib would reduce monocytosis and cell adhesion, and provide us with a tool to lower monocyte contribution to the plaque in early and late atherosclerosis.

## Methods

### Animal models

6-week-old female Apoe^−/−^ mice (B6.129P2-Apoe^tm1Unc^/J) were purchased from Charles River (Calco, Italy) and consumed a high cholesterol diet (1.25 % cholesterol; D12108 mod., Ssniff GmBH, Soest, Germany) ad libitum for 8 and 20 weeks, respectively, as indicated. Fostamatinib disodium (Astra Zeneca, UK) without carrier was incorporated at 0.3 % (w/w) into the diet as previously described [6]. 1 mg BrdU per mouse (BD Bioscience, San Jose, CA, USA) was injected intravenously 2 h before euthanasia. Intravital microscopy of mesenteric veins was performed 3 h after intraperitoneal stimulation with TNFα. Ly6C^high^ monocytes were mobilized into peripheral blood by intravenous injection of 300 ng CCL2 (R&D System, Minneapolis, MN, USA). 1 μm large green fluorescent beads (Fluoresbrite YG plain microspheres, Polysciences Inc., Eppelheim, Germany), diluted 1:4 in sterile PBS, were injected intravenously for in vivo cell labeling and tracking. Mice were housed under specific pathogen-free conditions and procedures approved by the Animal Care Committee of the University of Freiburg and the Regional Council (G-12/083).

### Histology

Aortic roots were embedded in OCT Tissue Tek (Sakura Finetek, Tokyo, Japan) for sectioning and staining with specific antibodies that are listed in the supplemental method section. Abdominal aortas were pinned for Oli-red O en face staining.

### Flow cytometry and cell sorting

Cells isolated from the blood, spleen, bone marrow and aorta were stained with specific antibodies and dyes that are listed in the supplemental method section. The APC Brdu Flow Kit detected intracellular BrdU incorporation (BD Bioscience, San Diego, CA, USA) according to the manufacturer’s instructions. Intracellular phospho-SYK^Tyr519/520^ (Cell Signaling Technology, Boston, MA, USA) was evaluated in fixed and permeabilized (ebioscience, San Jose, CA, USA) bone marrow and blood cells. Data were recorded on a BD Facs Canto II (BD Bioscience, San Diego, CA, USA). Monocytes and macrophages were sorted with a BD Facs Aria III (BD Bioscience, San Diego, CA, USA).

### Colony forming cell assay

Bone marrow cells were isolated from the femur and resuspended in Mouse Methylcellulose Complete Medium (R&D, Minneapolis, MN, USA) at 10^5^ cells/ml. Cells were stimulated with 10 ng/ml GM-CSF and 10 ng/ml IL3 (Peprotech, Rocky Hill, NJ, USA) in the presence of the vehicle DMSO 0.1 %, 0.1uM R406 in DMSO 0.1 % and 1μM R406 in 0.1 % DMSO, respectively, as indicated. Cells were incubated at 37 °C and 5 % CO_2_ for 5 days according to the manufacturer’s instructions and the number of colonies per 35 mm culture plate was counted under the microscope.

### Cell culture and transwell assay

Ly6C^high^ monocytes isolated from the blood and spleen were stimulated with 30 ng/ml M-CSF (Peprotech, Rocky Hill, NJ, USA) in normal culture media (RPMI-1640, 10 % FCS, NEAA, 1 % PenStrep) as indicated. Ly6C^high^ monocytes isolated from the bone marrow were differentiated into macrophages with 30 ng/ml M-CSF in normal culture media over 7 days and then stimulated with 10 μg/ml human DiI-medium oxidized LDL (Kalen Biomedical, Montgomery Village, MD, USA) for 16 h in a minimal medium (2 % FCS in RPMI without growth factor supplements). Foam cell formation was evaluated by microscopy and flow cytometry. Bone marrow monocytes, isolated with the EasySep Mouse Monocyte Isolation Kit (Stemcell Technologies, Cologne, Germany) according to the manufacturer’s instructions, were allowed to migrate for 3 h in a transwell chamber (2 × 10^5^ cells per well; Costar 6.5 mm Transwell inserts with 5 μm pore membrane, Corning, Kennebunk, ME, USA) stimulated by CCL2 20 ng/ml, CCL5 20 ng/ml (R&D System, Minneapolis, MN, USA) or BSA 20 ng/ml as control. Cells in the lower chambers were quantified with an automated cell counter (Z2 analyzer, Beckman Coulter, Inc., Brea, CA, USA).

### RNA isolation and real-time PCR

Cells were sorted into RLT/β-mercaptoethanol for subsequent RNA isolation using the RNeasy Micro Kit (Qiagen, Valencia, CA, USA) according to the manufacturer’s instructions. The Oviation PicSL WTA System V2 (NuGEN, San Carlos, CA, USA) was used for reverse transcription and cDNA amplification according to the manufacturer’s instructions. Quantitative TaqMan-PCR was run on a Bio-Rad CFX96 Touch Real-Time PCR System (Bio-Rad Laboratories, Hercules, CA, USA). The specific TaqMan probes used are listed in the supplemental method section.

### Statistics

Results are presented as mean ± SEM. Differences between 2 groups were evaluated by the unpaired Student’s *t* test if they passed the Kolmogorov–Smirnov normality test or otherwise by the non-parametric Mann–Whitney test as indicated. Differences between more than 2 groups were evaluated by Kruskal–Wallis test with Dunn’s multiple comparison test. *p* values ≤0.05 denote significant changes.

## Results

### SYK inhibition attenuates atherosclerotic plaque development in Apoe^−/−^ mice

**Fig. 1:**
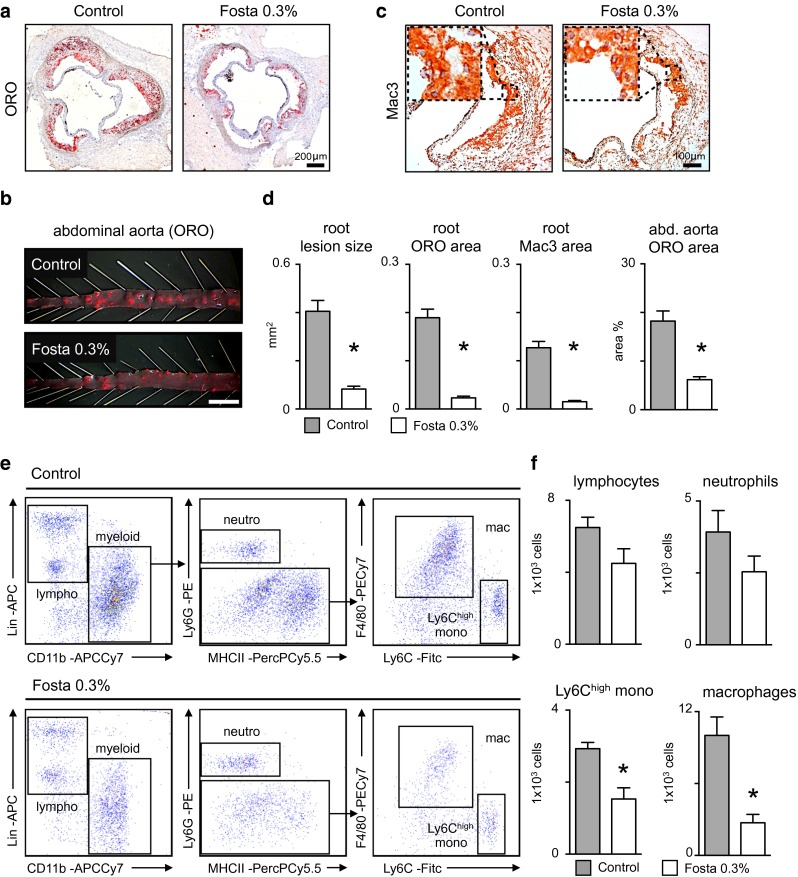
Fostamatinib reduces atheroma initiation in Apoe^−/−^ mice. **a** Representative Oil Red O (*ORO*) stainings of aortic root sections and (**b)** abdominal aortas from control and fostamatinib 0.3 % w/w (Fosta 0.3 %) treated Apoe^−/−^ mice after 8 weeks of high cholesterol diet (*HCD*). **c** Representative macrophage (*Mac3*) staining of aortic root sections with higher magnification from control and fostamatinib 0.3 % treated Apoe^−/−^ mice after 8 weeks of HCD. **d** Quantification of lesion size, lipid (ORO) and macrophage (Mac3) content in aortic root lesions (*n* = 14 per group) and lesion area in abdominal aortas (*n* = 8 per group) of control (*gray*) and fostamatinib 0.3 % (*white*) treated Apoe^−/−^ mice after 8 weeks of HCD. Results are presented as mean ± SEM. *p ≤ 0.05, *t* test. **e** Analysis of aortic plaque lesions by flow cytometry and (**f**) quantification of lymphocytes (*lympho*), myeloid cells, macrophages (*mac*), neutrophils (*neutro*) and Ly6C^high^ monocytes (*mono*) in control (*gray*) and fostamatinib 0.3 % (*white*) treated Apoe^−/−^ mice (*n* = 6 per group) after 8 weeks of HCD. Results are presented as mean ± SEM. *p ≤ 0.05, *t* test. *Lin* lineage cocktail with anti-CD3, anti-CD19, anti-NK1.1

6-week-old Apoe^−/−^ mice, still devoid of atherosclerosis, consumed a high cholesterol diet (HCD) supplemented with or without 0.3 % (w/w) SYK inhibitor fostamatinib for 8 weeks. At this point, we observed de novo plaque formation in the aortic root and abdominal aorta. Histologic analysis revealed that SYK inhibition markedly reduced overall lesion size, lipid and macrophage content in the aortic root and abdominal aorta, respectively (Fig. 1a–d), despite similar plasma cholesterol levels (Supplemental Table 1). Flow cytometric analysis of aortic tissue lysates confirmed a significant reduction in Ly6C^high^ monocyte and macrophage numbers (Fig. 1e, f).

### SYK inhibition reduces medullary and extramedullary myelopoiesis in atherosclerotic Apoe^−/−^ mice

**Fig. 2:**
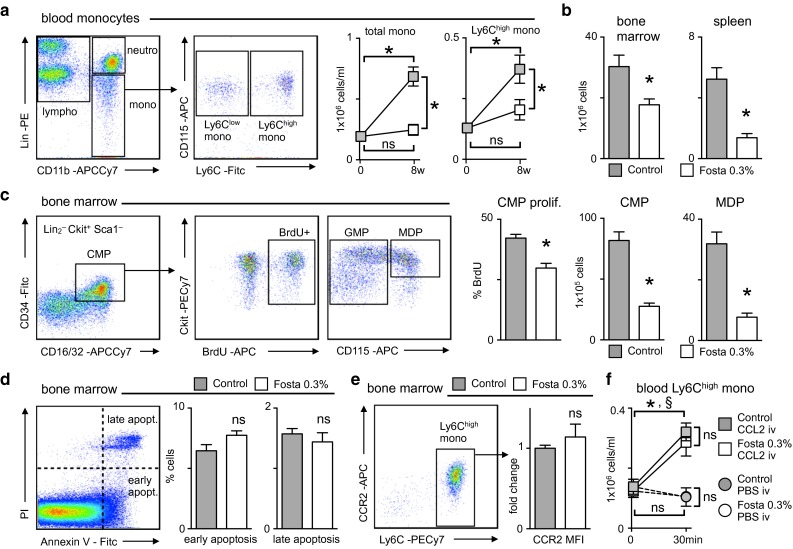
Fostamatinib inhibits monocytosis in hypercholesterolemic Apoe^−/−^ mice. **a** Identification and quantification of blood monocyte subsets by flow cytometry at baseline (*n* = 8) and after 8 weeks of HCD in control (*gray*) and fostamatinib 0.3 % (*white*) treated Apoe^−/−^ mice (*n* = 14 per group). Results are presented as mean ± SEM. *p ≤ 0.05. *ns* not significant if *p* > 0.05, *t* test. *Lin* lineage cocktail with anti-CD3, anti-CD19, anti-NK1.1, anti-Ly6G. **b** Quantification of Ly6C^high^ monocytes in the bone marrow and spleen of control (*gray*) and fostamatinib 0.3 % (*white*) treated Apoe^−/−^ mice after 8 weeks of HCD (*n* = 14 per group). Results are presented as mean ± SEM. *p ≤ 0.05, *t* test. **c** Identification and quantification of common myeloid progenitor (*CMP*) cell number and rate of proliferation as determined by bromodeoxyuridine (*BrdU*) incorporation. Identification and quantification of granulocyte macrophage (*GMP*) and macrophage dendritic cell progenitors (*MDP*) in the bone marrow of control (*gray*) and fostamatinib 0.3 % (*white*) treated Apoe^−/−^ mice (*n* = 10 per group) after 8 weeks of HCD. Results are presented as mean ± SEM. *p ≤ 0.05, *t* test. *Lin*_*2*_ lineage cocktail with anti-CD3, anti-CD90.2, anti-CD19, anti-NK1.1, anti-CD49b, anti-Gr-1, anti-CD11b, anti-CD11c, anti-IL7Ra. **d** Identification and quantification of early and late bone marrow cell apoptosis of control (*gray*) and fostamatinib 0.3 % (*white*) treated Apoe^−/−^ mice (*n* = 10 per group) after 8 weeks of HCD by Annexin V and PI staining. Results are presented as mean ± SEM. *ns* not significant if *p* > 0.05, *t* test. **e** Quantification of CCR2 mean fluorescence intensity on Ly6C^high^ bone marrow monocytes of control (*gray*) and fostamatinib 0.3 % (*white*) treated Apoe^−/−^ mice (*n* = 10 per group) after 8 weeks of HCD. Results are presented as mean ± SEM. *ns* not significant if *p* > 0.05, *t* test. **f** Apoe^−/−^ mice consumed a HCD with or without fostamatinib 0.3 % for 4 days, when peripheral monocyte numbers were still unaffected. Ly6C^high^ monocytes of control (*gray*) and fostamatinib 0.3 % (*white*) treated Apoe^−/−^ mice were counted in the blood before and 30 min after intravenous CCL2 (*n* = 6 per group; *squares*) or PBS (*n* = 4 per group; *circles*) administration. Results are presented as mean ± SEM. *p ≤ 0.05 within the control group and ^§^ *p* ≤ 0.05 within the fostamatinib group, Kruskal–Wallis test. *ns* not significant if *p* > 0.05

In accord with reduced cell counts in the aorta fostamatinib prevented the rise in circulating Ly6C^high^ monocytes associated with hypercholesterolemia and atherogenesis (Fig. 2a). We queried the possible mechanisms. First, Ly6C^high^ monocyte numbers failed to increase in the bone marrow and spleen after 8 weeks of HCD with fostamatinib intake (Fig. 2b) indicating hampered medullary and extramedullary myelopoiesis. Treatment with the SYK inhibitor lowered both the percentage of common myeloid progenitors (CMP) that incorporated BrdU and the frequency of their progeny, the macrophage dendritic cell progenitors (MDP), that give rise to monocytes, in the bone marrow and spleen (Fig. 2c, Supplemental Figure 1). Secondly, we found no signs of increased myelotoxicity with fostamatinib as assessed by Annexin V and PI staining (Fig. 2d). Lastly, fostamatinib-treated and control mice showed similar CCR2 expression levels on Ly6C^high^ monocytes in the bone marrow and no difference in their mobilization upon intravenous CCL2 administration (Fig. 2e, f). These data indicate that fostamatinib inhibited hypercholesterolemia-associated inflammatory monocyte production.

### SYK inhibition blocks GM-CSF/IL-3 stimulated myelopoiesis

**Fig. 3:**
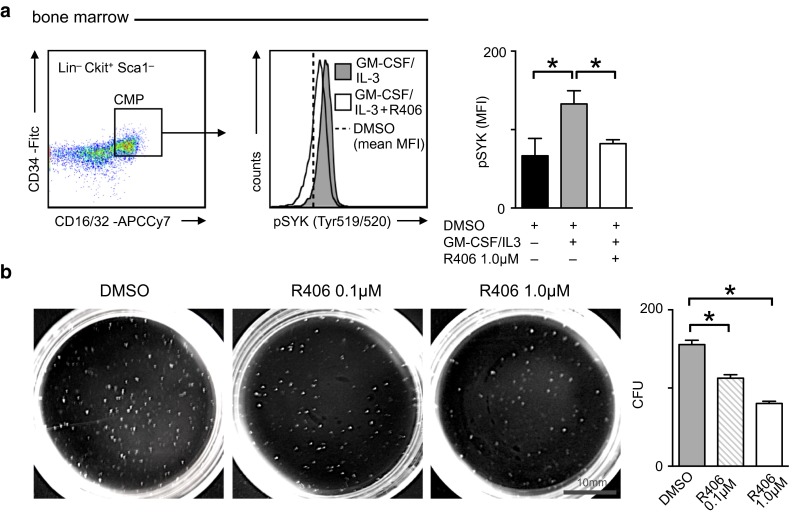
The monocytopoietic growth factors GM-CSF and IL3 activate SYK. **a** Quantification of SYK phosphorylation (Tyr519/520) in bone marrow common myeloid progenitor cells isolated from Apoe^−/−^ mice and stimulated with GM-CSF and IL3 for 10 min in the presence (*white*) or absence (*gray*) of SYK inhibitor R406 as determined by flow cytometry. Unstimulated DMSO controls are depicted as a *dashed line* and *black bar*, respectively (*n* = 3 duplicates per group). Results are presented as mean ± SEM. *p ≤ 0.05, Kruskal–Wallis test. *Lin* lineage cocktail with anti-CD3, anti-CD90.2, anti-CD19, anti-NK1.1, anti-CD49b, anti-Gr-1, anti-CD11b, anti-CD11c, anti-IL7Ra. **b** Colony forming unit (CFU) assay of bone marrow cells isolated from Apoe^−/−^ mice stimulated with GM-CSF and IL3 in the presence or absence (*gray*) of SYK inhibitor R406 (0.1 μM in *shaded*, 1.0 μM in *white*). Results are presented as mean ± SEM for *n* = 4 duplicates per group. *p ≤ 0.05, Kruskal–Wallis test

Increased medullary and extramedullary monocyte production in Apoe^−/−^ mice results from impaired cholesterol efflux and heightened surface expression of the common beta subunit of the GM-CSF/IL-3 receptor (CBS) on progenitor cells [10, 12, 19]. GM-CSF and IL-3 stimulate myeloid progenitor cell proliferation and SYK binds to the CBS for signal transduction [18]. We observed SYK phosphorylation at Tyr^519/520^ associated with kinase activation in bone marrow CMP of Apoe^−/−^ mice in response to stimulation with GM-CSF and IL-3. R406, the active metabolite of fostamatinib, limited GM-CSF/IL-3 induced SYK autophosphorylation (Fig. 3a). SYK inhibition by R406 reduced colony formation by bone marrow cells stimulated with GM-CSF and IL-3 in line with our in vivo data (Fig. 3b).

### SYK inhibition attenuates monocyte infiltration and differentiation but not macrophage egress from atherosclerotic lesions

**Fig. 4:**
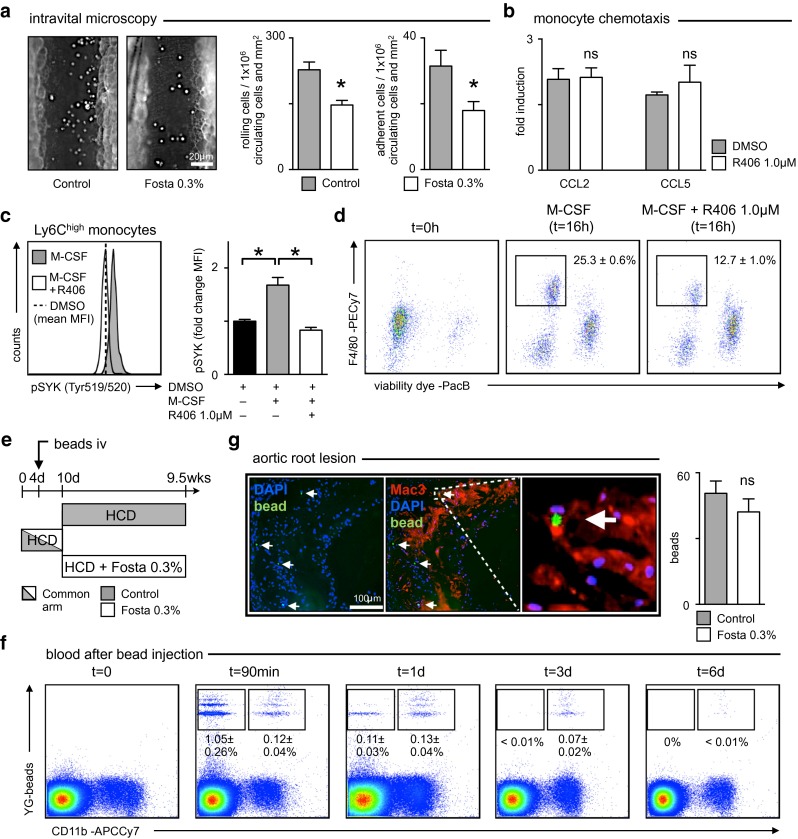
Fostamatinib inhibits de novo macrophage accumulation but not egress from atherosclerotic lesions. **a** Quantification of rolling and adherent leukocytes by intravital microscopy of mesenteric veins in control (*gray*) and fostamatinib 0.3 % (*white*) treated Apoe^−/−^ mice (4 days of HCD) 3 h after intraperitoneal TNFα administration. Results are normalized to individual peripheral cell counts and the vessel segment areas analyzed, and are presented as mean ± SEM, *n* > 50 vessel segments per group. *p ≤ 0.05, *t* test. **b** Quantification of CCL2 and CCL5 stimulated monocyte migration over BSA in a transwell chamber. Results are presented as mean ± SEM, *n* = 5 per group. *p ≤ 0.05, *t* test. **c** Quantification of SYK phosphorylation (Tyr519/520) in Ly6C^high^ blood monocytes of Apoe^−/−^ mice stimulated with M-CSF for 10 min in the presence (*white*) or absence (*gray*) of SYK inhibitor R406 as determined by flow cytometry. Unstimulated DMSO controls are depicted as a *dashed line* and *black bar*, respectively (*n* = 3 duplicates per group). Results are presented as mean ± SEM. *p ≤ 0.05, Kruskal–Wallis test. **d** Ly6C^high^ monocytes isolated from the blood and spleen (*t* = 0 h) were stimulated with M-CSF in the presence or absence of SYK inhibitor R406 for 16 h. The fraction of viable cells that upregulated macrophage marker F4/80 was quantified by flow cytometry. Results are presented as mean ± SEM, *n* = 5 per group, *p* < 0.01, *t* test. **e** Treatment scheme of Apoe^−/−^ mice with HCD for 10 days (bicolored, common) before randomization to fostamatinib 0.3 % (*white*) or continued HCD (*gray*) for another 8 weeks. Fluorescent beads were injected intravenously on day 4. **f** Quantification of fluorescent beads freely circulating or ingested by CD11b^+^ myeloid cells in the blood at indicated time points after intravenous administration. Results are presented as mean ± SEM, *n* = 3. **g** Identification and quantification of fluorescent beads in aortic root lesions after 9.5 weeks of HCD in control (*gray*) and fostamatinib 0.3 % (*white*) treated Apoe^−/−^ mice (*n* = 8 per group). Representative immunohistologic staining for macrophages (*Mac3*), nuclei (*DAPI*) and fluorescent beads (*green*) on the left and quantification of beads within lesions of 8 adjacent aortic root sections per mouse. Results are presented as mean ± SEM. *p ≤ 0.05, *t* test

We next studied possible local effects of fostamatinib on lesional macrophage accumulation apart from monocyte production and mobilization. Consumption of HCD supplemented with 0.3 % fostamatinib for 4 days did not lower the number of leukocytes in the circulation but reduced their rolling along and adhesion to the intimal surface as assessed by intravital microscopy of TNFα stimulated mesenteric veins (Fig. 4a). SYK inhibition, however, did not interfere with CCL2 and CCL5 directed monocyte migration in a transwell chamber (Fig. 4b). The reduced number of lesional macrophages observed in fostamatinib-treated Apoe^−/−^ mice (Fig. 1e, f) may not only result from reduced monocyte availability inside the lesion but also from impaired monocyte to macrophage differentiation or increased macrophage egress. Monocyte to macrophage differentiation depends on M-CSF. Stimulation of mature Ly6C^high^ monocytes isolated from the blood and spleens of Apoe^−/−^ mice with M-CSF induced SYK activation and phosphorylation at Tyr^519/520^ that was abrogated by R406 (Fig. 4c). Consequently, overnight culture of sorted Ly6C^high^ monocytes with M-CSF and SYK inhibitor R406 blocked their differentiation into live F4/80^high^ macrophage-like cells (Fig. 4d). To evaluate macrophage egress from atherosclerotic lesions we injected fluorescent beads, as previously described [11], into young Apoe^−/−^ mice that had just been placed on HCD 4 days previously (Fig. 4e). Within 6 days myeloid cells had cleared the blood from circulating beads and infiltrated the nascent plaques (Fig. 4f). Fostamatinib treatment was started at this time point in one group while the other group continued on HCD for another 8 weeks, thus avoiding any influence of the drug on the initial cell mediated accumulation of beads in lesions (Fig. 4e). The beads are non-degradable and can only leave the lesions within emigrating phagocytes [11]. Therefore, the number of beads remaining in the lesion inversely correlates with macrophage egress. 8 weeks of fostamatinib treatment did not change the number of beads in lesions but still reduced the lesional macrophage content (Fig. 4g, Supplemental Figure 2). We concluded that SYK inhibition mainly limited the number of infiltrating monocytes and their differentiation but not macrophage emigration from atherosclerotic lesions.

### SYK inhibition exerts subtle effects on macrophage foam cell formation and inflammation

**Fig. 5:**
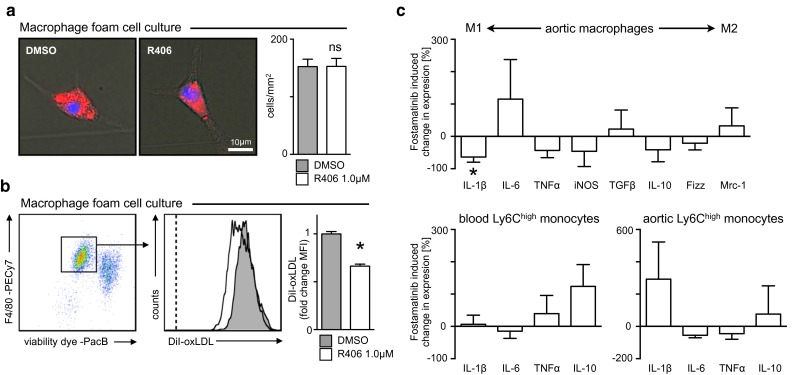
Fostamatinib exerts subtle effects on macrophage foam cell formation and inflammation. **a**, **b** Ly6C^high^ monocytes were stimulated with M-CSF to generate macrophages in culture before incubation with DiI-oxLDL under starving conditions in the presence (*white*) or absence (*gray*) of SYK inhibitor R406. **a** Identification and quantification of macrophage foam cells by immunofluorescence microscopy. Representative images are shown on the *left*. Results are presented on the *right* as mean ± SEM. *p ≤ 0.05, *t* test. **b** Quantification of DiI-oxLDL accumulation by flow cytometry. Results are presented as mean ± SEM. *p ≤ 0.05, Mann–Whitney test. **c** Gene expression profiling of macrophages and Ly6C^high^ monocytes isolated from atherosclerotic aortas and blood, respectively, after 8 weeks of HCD in control and fostamatinib 0.3 % treated Apoe^−/−^ mice (*n* = 6 per group). Results are presented as mean ± SEM percent change of marker expression in fostamatinib-treated mice compared to controls. *p ≤ 0.05, *t* test. Other changes are statistically not significant

Macrophages accumulate lipids in atherosclerotic lesions and develop into foam cells. We tested if SYK inhibition affected foam cell formation. To this end, we generated Ly6C^high^ monocyte derived macrophages that were incubated with DiI-labeled oxidized low density lipoprotein (DiI-oxLDL) overnight in the presence or absence of SYK inhibitor R406. The number of macrophages in culture remained unchanged, and all viable macrophages ingested oxLDL, though SYK inhibition reduced the mean fluorescence intensity of DiI by one-third (Fig. 5a, b), similar to what had previously been observed with other lipoprotein modifications [2]. We next asked whether fostamatinib altered the inflammatory phenotype of monocytes and macrophages in the context of atherosclerosis. Macrophages and Ly6C^high^ monocytes were sorted from atherosclerotic aortas alongside Ly6C^high^ monocytes from the blood after 8 weeks of HCD with our without fostamatinib. RNA was isolated for gene expression analysis. Overall, fostamatinib exerted rather subtle and fluctuating changes of the inflammatory profile in all cell types tested, except for significantly reduced (pro-)IL-1β expression by macrophages (Fig. 5c).

### SYK inhibition fails to prevent plaque progression

**Fig. 6:**
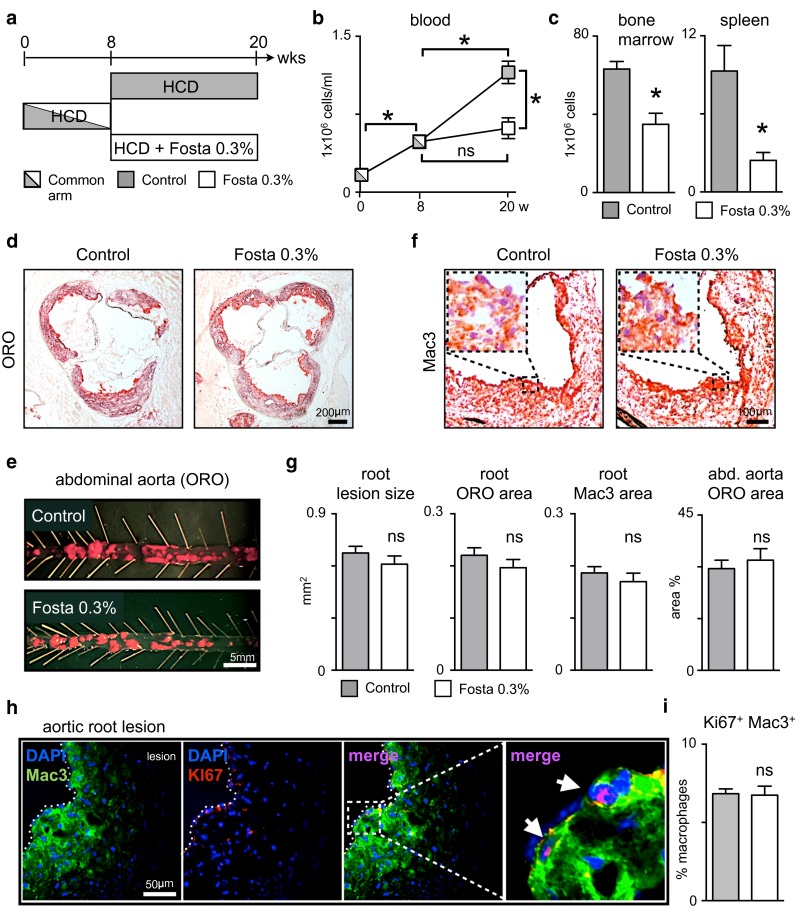
Continued lesion progression despite fostamatinib treatment with reduced monocytosis in established atherosclerosis. **a** Treatment scheme of Apoe^−/−^ mice with Fosta 0.3 % (*white*) or continued HCD (*gray*) over 12 weeks after a prior 8 week HCD feeding period to establish plaque development (bicolored, common). **b** Quantification of blood Ly6C^high^ monocytes by flow cytometry at baseline, after 8 weeks of HCD (bicolored, common) and after an additional 12 weeks of HCD with (*white*) or without (*gray*) Fosta 0.3 % supplementation (*n* = 18 per group). Results are presented as mean ± SEM. *p ≤ 0.05. *ns* not significant if *p* > 0.05. **c** Quantification of Ly6C^high^ monocytes in the bone marrow and spleen of control (*gray*) and Fosta 0.3 % (*white*) treated Apoe^−/−^ mice after 8 + 12 weeks of HCD (*n* = 12 per group). Results are presented as mean ± SEM. *p ≤ 0.05. **d** Representative Oil Red O (*ORO*) stainings of aortic root sections and (**e**) abdominal aortas from control and Fosta 0.3 % treated Apoe^−/−^ mice after 8 + 12 weeks of HCD. **f** Representative macrophage (*Mac3*) staining of aortic root sections with higher magnification from control and fostamatinib 0.3 % treated Apoe^−/−^ mice after 8 + 12 weeks of HCD. **g** Quantification of lesion size, lipid (ORO) and macrophage (Mac3) content in aortic root lesions and of lesion area in abdominal aortas (*n* = 18 per group) of control (*gray*) and fostamatinib 0.3 % (*white*) treated Apoe^−/−^ mice after 8 + 12 weeks of HCD. Results are presented as mean ± SEM. *ns* not significant if *p* > 0.05. **h** Representative immunohistologic staining for macrophages (Mac3), nuclei (*DAPI*) and proliferation marker Ki67 of aortic root sections from control and Fosta 0.3 % treated Apoe^−/−^ mice after 8 + 12 weeks of HCD. **i** Percentage of KI67 + proliferating lesional macrophages (*n* ≥ 50 visual fields per group). Results are presented as mean ± SEM. *ns* not significant if *p* > 0.05

Next, we asked whether SYK inhibition by fostamatinib could modulate progression of established plaques. Apoe^−/−^ mice consumed a HCD for 8 weeks to allow plaque formation throughout the aorta (common group). Then mice were randomized to either HCD alone or HCD supplemented with 0.3 % (w/w) fostamatinib for another 12 weeks (Fig. 6a). Again, treatment with fostamatinib abrogated the continuous rise of Ly6C^high^ monocytes in the blood, bone marrow and spleen (Fig. 6b, c). In contrast, fostamatinib intake did not alter lesion progression in the root and abdominal aorta. Lesional lipid and macrophage contents did not differ between groups (Fig. 6d–g). To address the dissociation between circulating monocytes and lesional macrophage accumulation we quantified macrophage proliferation and cell death in aortic root sections. Fostamatinib treatment did not change the percentage of proliferating Ki67^+^ and apoptotic TUNEL^+^ macrophages in the plaque (Fig. 6h, i, Supplemental Figure 3).

## Discussion

**Fig. 7:**
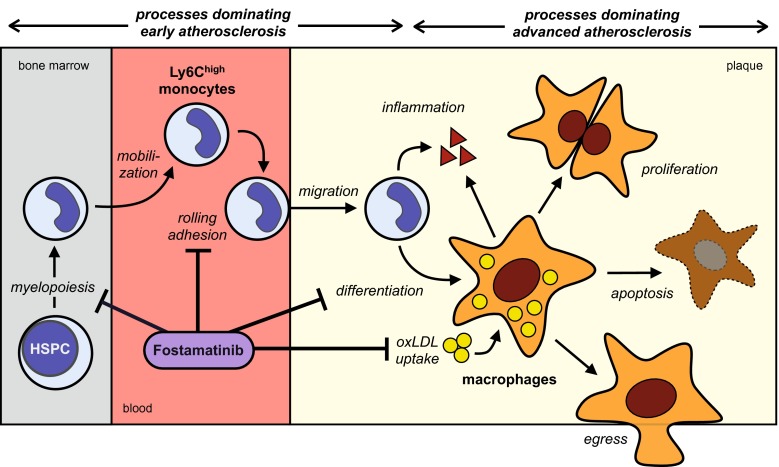
A model of selective fostamatinib effects on monocyte/macrophage dynamics and function in atherosclerosis

We show here that SYK inhibition by fostamatinib differentially interfered with the turnover and accumulation of monocytes and macrophages in atherosclerotic lesions on multiple levels. Fostamatinib impaired hypercholesterolemia-associated monocytosis stimulated by GM-CSF and IL-3 in Apoe^−/−^ mice. In concert with reduced integrin-mediated cell adhesion [6, 9, 16] and hampered M-CSF-dependent macrophage differentiation, fostamatinib potently inhibited the early accumulation of lesional macrophages and the development of new atherosclerotic lesions by more than 70 %. Chemoattraction mediating monocyte/macrophage mobilization and migration involves G-protein-coupled receptor signaling [8, 20] independent of SYK and, therefore, may have remained unaffected. Our findings agree with the concept that macrophages in early atherosclerosis directly derive from infiltrating Ly6C^high^ monocytes [7]. In established lesions, however, local foam cell proliferation rather than monocyte recruitment and differentiation dominates macrophage turnover [13]. SYK inhibition by fostamatinib, when started after lesions had already formed, still reduced Ly6C^high^ monocytosis by about 50 %. Fewer monocytes, however, did not translate into reduced macrophage accumulation in advanced lesions. In fact, lesions continued to grow throughout the aorta by more than 50 % even with fostamatinib treatment. Half the amount of circulating Ly6C^high^ monocytes might still suffice to sustain the macrophage pool in atherosclerotic lesions of fostamatinib-treated mice. We propose, however, that the failure to influence intraplaque macrophage proliferation, or cell death or egress rendered fostamatinib treatment ineffective in established disease (Fig. 7). While GM-CSF/IL-3 stimulate myeloid progenitor cell proliferation via SYK, macrophage proliferation in more advanced lesions does not depend on GM-CSF/IL-3 signaling [13]. Rheumatoid arthritis and atherosclerosis share important common pathogenic mechanisms, and GM-CSF drives monocytosis in experimental rheumatoid arthritis [3]. When rheumatoid patients are treated with fostamatinib blood monocyte levels drop by 36 % [5].

Our study indicates that the treatment of atherosclerosis may require disease stage specific approaches. Interventions directed against monocyte production and recruitment may limit early lesion formation or acute aggravation. In advanced lesions, however, when local proliferation dominates macrophage accumulation, targeting cell division itself or the respective stimulators may prove more effective. This concept, if applicable to humans, has major clinical implications as patients with atherosclerosis usually become symptomatic only after plaques have already formed.

## Electronic supplementary material

Supplementary material 1 (PDF 270 kb)
