# Supplementary material for: Atheroprotection through SYK inhibition fails in established disease when local macrophage proliferation dominates lesion progression
Source: Basic Res Cardiol. 2016 Feb 18;111:20. doi: 10.1007/s00395-016-0535-8 (PMC4759214; doi:10.1007/s00395-016-0535-8)
Supplement: Supplementary file 1 — Supplementary material 1 (PDF 270 kb) [file 395_2016_535_MOESM1_ESM.pdf]

Supplemental Material

Supplemental Methods

Study design. This study investigates SYK inhibition by Fostamatinib in early and advanced atherosclerosis. Fostamatinib disodium (Astra Zeneca, UK) without carrier was incorporated at 0.3% (w/w) into high cholesterol diet (HCD) pellets (1,25% cholesterol; D12108 mod., Ssniff GmbH, Soest, Germany) as previously described [7]. Different color dyes were used to mark control and drug diet. To study SYK inhibition in early atherosclerosis 6 week old female Apoe$^{-/-}$ mice consumed a HCD ad libitum for 8 weeks with or without Fostamatinib. To study effects in established atherosclerosis 6 week old female Apoe$^{-/-}$ mice consumed a HCD for 8 weeks before being randomized to HCD supplemented with fostamatinib or HCD alone for another 12 weeks.

Animal procedures and tissue processing. Mice were anesthetized with isofluran for retroorbital bleedings, intravenous injection of 1μm Fluoresbrite YG microspheres (230μl of a 1:4 dilution in sterile PBS, Polysciences Inc., Eppelheim, Germany), intravenous injection of 300ng CCL2 (R&D System, Minneapolis, MN, USA) and intravenous injections of BrdU 1mg/mouse (0.1 ml, BD Bioscience, San Jose, CA, USA) 2 hours prior to sacrifice. For intravital microscopy mice were stimulated by intraperitoneal injection of 10mg/kg TNFα (R&D System, Minneapolis, MN, USA) 3 hours before anesthesia by intraperitoneal injection of 87.5mg/kg Ketamine plus 12.5mg/kg Xylazine. 50μl of rhodamine 6G (1mg/ml) were injected intravenously to label leukocytes before the peritoneal cavity was cut open and the mesentery was carefully spread out on a glas cover slide thereby exposing mesenteric veins. Leukocyte rolling and adhesion within 100μm long and 50-80μm wide vein segments was recorded over 30sec with an AxioScope Vario and analyzed with the Axiovision Software (Carl Zeis Inc., Oberkochen, Germany). Mice were euthanized with CO2. Spleens, femurs and aortas were excised after vascular perfusion with 10ml NaCl 0.9%. Minced spleen and flushed bone marrow suspensions were strained through a 40 μm-nylon mesh (BD Biosciences). Blood and splenic cell suspensions were lysed in RBC lysis buffer (Biolegend, San Diego, Ca, USA). The aortic sinus was embedded in OCT (Sakura Tissue Tek, Torrance, CA), abdominal aortas were fixed in 10% formalin for en face staining. In order to obtain aortic cell suspensions for flow cytometry aortas were excised and opened longitudinally for pinning and brief enzymatic digestion of the intima. Plaques scraped and picked from the thoracoabdominal aorta and the plaque laden aortic root plus ascending aorta were collected, minced and digested in 450 U/ml collagenase I, 125 U/ml collagenase XI, 60 U/ml DNase I, 60 U/ml hyaluronidase (Sigma-Aldrich, St. Louis, MO, USA) for 45minutes at 37°C while shaking (750rpm). Cells were counted with a Hemocytometer (Neubauer Chamber).

Flow Cytometry. Cell suspensions were stained in PBS w/o Ca/Mg supplemented with sterile 1% FBS and 0.5% BSA. The following monoclonal antibodies were used for flow cytometric analysis:
anti-Ly6C (clone AL-21, BD Biosciences), anti-CD34 (clone RAM34, BD Biosciences), anti-CD45.2 (clone 104, eBioscience), anti-CD3e (clone 145-2C11, eBioscience), anti-CD19 (clone eBio1D3, eBioscience), anti-F4/80 (clone BM8, Biolegend), anti-CD49b (clone DX5, eBioscience), anti-NK1.1 (clone PK136, eBiosciences), anti-Ly6G (clone 1A8, Biolegend), anti-Gr-1 (clone RB6-8C5,

eBioscience), anti-CD11b (clone M1/70, BD Biosciences), anti-CD115 (clone AFS98, eBioscience), anti-Ckit (clone 2B8, Biolegend), anti-IL7Rα (clone A7R34, Biolegend), anti-Sca1 (clone Ly6A/E, Biolegend), CCR2 (FAB5538A, R&D), CD16/32 (clone 2.4G2, BD Bioscience), anti-BrdU (BD Bioscience APC BrdU Flow Kit), Annexing V (BD Bioscience FITC Annexing V Apoptosis Detection Kit), anti-pSyk$^{mY519/520;hY525/526}$ (C87C1 rabbit mAb, 2710S, Cell Signaling Technology, Danvers, MA, USA) and Fab2-PE Conjugate (Cell Signaling Technology). For intracellular BrdU staining cells were fixed and permeabilized with BD Cytofix/Cytoperm (BD Biosciences) according to the manufacturer's instructions. For intracellular pSyk detection bone marrow cells were incubated at 37°C, 5% CO2 in serum free RPMI-1640 medium 0.1% DMSO with or without 1μM R406 for 2 hours. Cells were stimulated with 100ng/ml GM-CSF and 100ng/ml IL3 (Peprotech) for 10 minutes before fixation and permeabilization according to the manufacturer's instructions (eBioscience Fixation and Permeabilization Buffer Kit, 88-8823-88).

Data were analyzed with FlowJo. Specifically, macrophages were identified as $CD45^+$, Lin^- (Lin = CD3, CD19, NK1.1), $CD11b^+$, $F4/80^{high}$, $Ly6C^{low}$ cells. Monocytes were identified as $CD45^+$, Lin^-, $CD11b^+$, $CD115^+$ cells with high or low Ly6C expression. Neutrophils were identified as $CD45^+$, $L6G^+$, $CD11b^+$, $Ly6C^{int}$, SSC^{high} cells. Common myeloid progenitors (CMP) were identified as Lin_2^- (Lin_2 = CD3, CD90.2, CD19, NK1.1, CD49b, Gr-1, CD11b, CD11c, IL7Rα), $ckit^+$, $Sca1^-$, $CD34^{high}$, $CD16/32^{high}$ cells with macrophage dendritic cell progenitors (MDP) being $ckit^{high}$ $CD115^+$.

TaqMan PCR. Quantitative PCR was performed on a Bio-Rad CFX96 Touch Real-Time PCR System (Bio-Rad Laboratories, Hercules, CA, USA) using 40ng of amplified cDNA per sample and target, when possible, and the TaqMan Fast Advanced Master Mix according to the manufacturer's instructions. The following TaqMan gene expression assays were used: IL-1β Mm01336189_m1, IL-6 Mm00446190_m1, IL-10 Mm00439614_m1, TNFα Mm00443260_g1, Nos2 Mm00440502_m1, TGFβ1 Mm01178820_m1, Retnla/Fizz Mm00445109_m1, Mrc1/CD206 Mm00485148_m1, housekeeping gene β-actin 4351315E. Data were quantified with the $2^{-\Delta Ct}$ method.

Cholesterol assay. Total cholesterol levels were measured in heparinized plasma after a > 6 hour fasting period using the Lab Assay Cholesterol (code 294-65801, Wako) according to the manufacturer's instructions. The colorimetric assay was measured with a SpectraMAX Plus at 600nm wavelength (Serial P02918, Molecular Devices).

Histology. Frozen sections of the aortic roots were stained for lipids with Oil-Red-O (Sigma Aldrich, cat# 00625-25G) and macrophages with anti-Mac3 (rat anti-mouse, BD Pharmingen, clone M3/84, cat#553322). For immunofluorescent co-staining we used the Ki-67 (clone SP6, Abcam, #ab16667) and TUNEL kits (Promega, #G3250) according to the manufacturer's instructions. Abdominal aortas were fixed in 10% buffered formalin solution (Th. Geyer GmbH & Co, #2137) over night and washed in PBS prior to incubation with 85% propylene glycol for 2 minutes (Carl Roth, #0340.3). Aortas were stained with 0.5% Oil Red O for 4 hours at room temperature and washed with 85% propylene glycol and PBS. External fat was removed before aortas were cut longitudinally and pinned down on a silicon-elastomere plate (Factor II, cat#2186). En face Oil Red O staining and washing was repeated. Lesion and

macrophage areas were quantified by blinded investigators with a computer-assisted image analysis software (Image-Pro Plus 5.1.2 Media Cybernetics).

Supplemental Figures and Table

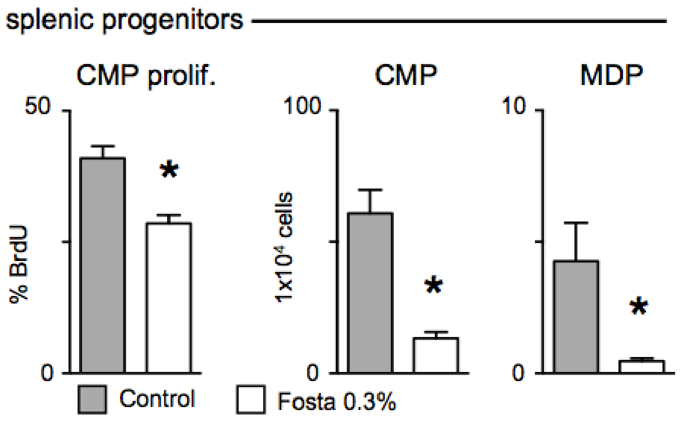

Supplemental Fig. 1 Fostamatinib inhibits hypercholesterolemia-associated extramedullary myelopoiesis.
Quantification of common myeloid progenitor (CMP) cell number and rate of proliferation as determined by BrdU incorporation as well as the number of macrophage dendritic cell progenitors (MDP) in the spleens of control (grey) and fostamatinib 0.3% (white) treated Apoe$^{-/-}$ mice (n=10 per group) after 8 weeks of HCD. Results are presented as mean±SEM. *p≤0.05.

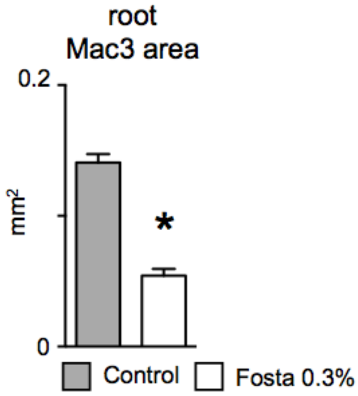

Supplemental Fig. 2 Fostamatinib lowers lesional macrophage content after fluorescent bead administration.
Apoe$^{-/-}$ mice received fluorescend beads intravenously on day 4 after the start of HCD and were randomized to continued HCD with or without fostamatinib 0.3% on day 10. Mice were sacrificed 8 weeks thereafter for histologic analysis of aortic root lesions. Quantification of macrophage (Mac3) content in aortic root lesions of control (grey) and fostamatinib 0.3% (white) treated Apoe$^{-/-}$ mice (n=8 per group). Results are presented as mean±SEM. *p≤0.05, t-test.

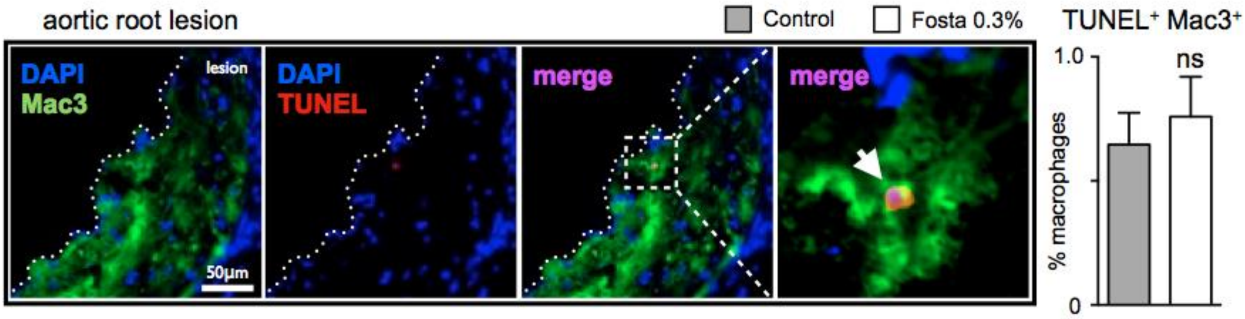

Supplemental Fig. 3 Fostamatinib does not affect lesional macrophage death. Representative immunohistologic staining for macrophages (Mac3), nuclei (DAPI) and cell death (TUNEL) of control and fostamatinib 0.3% treated Apoe$^{-/-}$ mice after 8+12 weeks of HCD. Results are presented as mean±SEM. ns, not significant if p>0.05.

Time	Control	Fosta 0.3%	p
8w	17.1 ± 0.7	16.1 ± 1.6	0.57
8+12w	16.3 ± 1.9	18.9 ± 2.4	0.40

Supplemental Table 1. Plasma cholesterol levels. Total cholesterol (mmol/l) was measured in the plasma of n=8 Apoe$^{-/-}$ mice per group after 8 weeks of HCD with or without fostamatinib 0.3% supplementation and after 8 weeks of HCD followed by 12 weeks of HCD with or without fostamatinib 0.3% supplementation, respectively. P-values for Student's t-test are given for each time point.
